# Causal inference in health and disease: a review of the principles and applications of Mendelian randomization

**DOI:** 10.1093/jbmr/zjae136

**Published:** 2024-08-21

**Authors:** Catherine E Lovegrove, Sarah A Howles, Dominic Furniss, Michael V Holmes

**Affiliations:** Nuffield Department of Surgical Sciences, University of Oxford, Oxford OX3 9DU, United Kingdom; Nuffield Department of Surgical Sciences, University of Oxford, Oxford OX3 9DU, United Kingdom; Nuffield Department of Orthopaedics, Rheumatology and Musculoskeletal Sciences, University of Oxford, Oxford OX3 7LD, United Kingdom; Medical Research Council, Integrative Epidemiology Unit, University of Bristol, Bristol BS8 2BN, United Kingdom

**Keywords:** epidemiology, genetic association studies, human association studies, mendelian randomization, therapeutics

## Abstract

Mendelian randomization (MR) is a genetic epidemiological technique that uses genetic variation to infer causal relationships between modifiable exposures and outcome variables. Conventional observational epidemiological studies are subject to bias from a range of sources; MR analyses can offer an advantage in that they are less prone to bias as they use genetic variants inherited at conception as “instrumental variables”, which are proxies of an exposure. However, as with all research tools, MR studies must be carefully designed to yield valuable insights into causal relationships between exposures and outcomes, and to avoid biased or misleading results that undermine the validity of the causal inferences drawn from the study. In this review, we outline Mendel’s laws of inheritance, the assumptions and principles that underlie MR, MR study designs and methods, and how MR analyses can be applied and reported. Using the example of serum phosphate concentrations on liability to kidney stone disease we illustrate how MR estimates may be visualized and, finally, we contextualize MR in bone and mineral research including exemplifying how this technique could be employed to inform clinical studies and future guidelines concerning BMD and fracture risk. This review provides a framework to enhance understanding of how MR may be used to triangulate evidence and progress research in bone and mineral metabolism as we strive to infer causal effects in health and disease.

## Introduction

**Figure 1:**
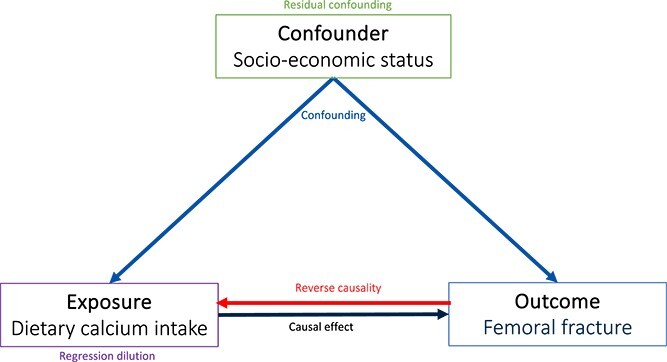
**Pitfalls in observational studies.** Confounding variable affecting exposure and outcome: measured confounders can be accounted for in multivariable analyses; unmeasured confounders can cause unreliable results. Residual confounding: confounding that persists despite adjusting for measured confounders. Reverse causality: possible effect of outcome on exposure, often neglected. Regression dilution bias: random errors in measurement causing bias in estimated association with outcome.

Defining disease-causing pathways is of central importance in medical research and can facilitate interventions to improve health. Conventional observational epidemiological approaches can allude to associations between exposures and outcomes, but are subject to limitations including the inability to detect the direction of causal relationships, bias from measurement error, or residual confounding from external factors that are not fully accounted for (Figure 1).^1^^,^^2^ These limitations may lead to erroneous conclusions regarding the nature of the relationship between an exposure and outcome. Well-designed randomized control trials (RCTs) are widely considered the “gold standard” approach to overcoming these problems to ascertain causal relationships,^3^ however not all research questions are suited to an RCT, for example where the outcome of interest is rare or takes a long time to manifest. Even when a hypothesis is amenable to testing in an RCT, there are practical barriers to conducting such a study. Furthermore, in cases where an exposure is known to be harmful, such as obesity, an RCT that randomizes a group of individuals to interventions aiming to establish a BMI over 30 kg/m^2^ would be unethical.^4^^,^^5^

**Figure 2:**
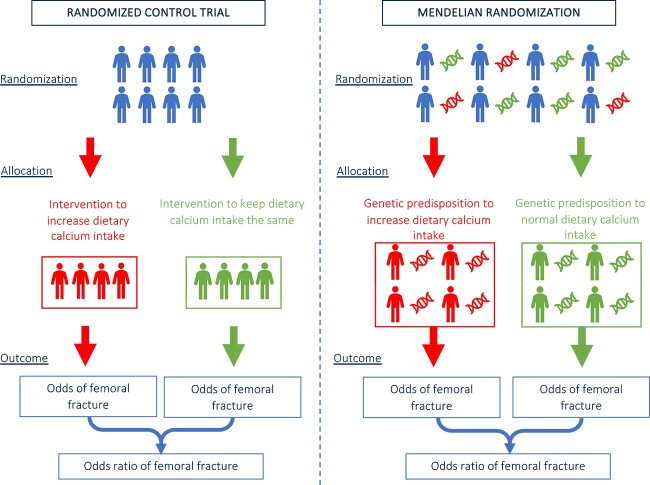
**Analogy of an RCT and MR.** MR leverages naturally occurring genetic variations as “instrumental variables” to mimic the random assignment of exposures, like random allocation in an RCT. This genetic randomization helps address confounding factors and strengthens causal inference when investigating the relationships between exposures, outcomes, and potential interventions.

Mendelian randomization (MR) is an epidemiological technique that aims to overcome the limitations of conventional epidemiological studies, and the practical difficulties associated with an RCT, by leveraging genetic variation to identify causal effects between an exposure and outcome.^6^ An analogy can be drawn between MR and an RCT in that MR exploits genetic alleles to allocate individuals to a “study arm” (Figure 2).^7^ The analogy has some limitations and cannot fully capture the intricacies of identifying causal effects, however it can be a helpful framework help conceptualize MR. Using genetic alleles, inherited at conception, to produce an unbiased proxy of an exposure trait, an “instrumental variable” (IV, Table 1), helps to mitigate against possible effects from environmental confounders (Table 1).^8–11^ Researchers commonly identify genetic variants associated with exposures and outcomes to use in MR studies from genome-wide association studies (GWAS); the effect on an outcome per unit increase in a genetically-predicted continuous exposure or the occurrence of an outcome in these genetically-predefined groups is subsequently assessed.

**Table 1:** Terminology in MR studies.

**Term**	**Definition**
**Confounder**	A variable associated with the exposure and the outcome that can induce a biased relationship between an exposure and outcome. This can be potentially accounted for in multivariable analyses; however, if overlooked, it can result in unreliable results
**Horizontal pleiotropy**	A genetic variant affects the outcome through traits independent of the hypothesized exposure
**Instrumental variable**	A genetic proxy for an exposure variable comprising genetic variants that are robustly associated with that variable
**Linkage disequilibrium**	A non-random distribution of genetic variants in the population due to their physical proximity on chromosomes causing them to be inherited together
**Mediator**	An intermediate variable through which an exposure affects an outcome
**One-sample MR**	An MR analysis that derives an IV exposure from the same dataset in which it conducts the MR analysis with the outcome
**Regression dilution**	Random errors in measurement of the exposure leading to bias toward the null in the estimated association of an exposure with an outcome
**Residual confounding**	Confounding that persists despite adjusting for measured confounders
**Reverse causality**	An effect of an outcome on an exposure often leading to full, or partial, misattribution of causality in the incorrect direction
**Two-sample MR**	An MR analysis that uses an exposure IV and an outcome IV derived from 2 separate datasets
**Vertical pleiotropy**	A genetic variant influences multiple traits (including the outcome variable of interest) through 1 causal pathway
**Weak instrument bias**	Bias arising from an IV that is weakly associated with the exposure variable, thus violating the “relevance” assumption

Abbreviations: IV, instrumental variable; MR, Mendelian randomization

MR studies have become increasingly common likely, in part, due to their potential to elucidate causal relationships and the widespread availability of GWAS data.^12^^,^^13^ Despite thousands of MR studies being published annually, many are unreliable as they don’t meet accepted standards.^14^^,^^15^ Just as an RCT must be carefully executed to avoid bias, meticulous design of MR studies is essential to ensure that analyses reliably evaluate the presence or absence of causal effects of an exposure on an outcome.^16^ Before using MR, researchers should understand its advantages and pitfalls. They ought also to have a clear, relevant, biologically plausible hypothesis with a strong rationale for using this technique. With the burgeoning popularity of MR in the scientific literature, this review aims to provide the readership with the tools to understand, critically appraise, and design MR studies.

## Assumptions and principles of MR

**Figure 3:**
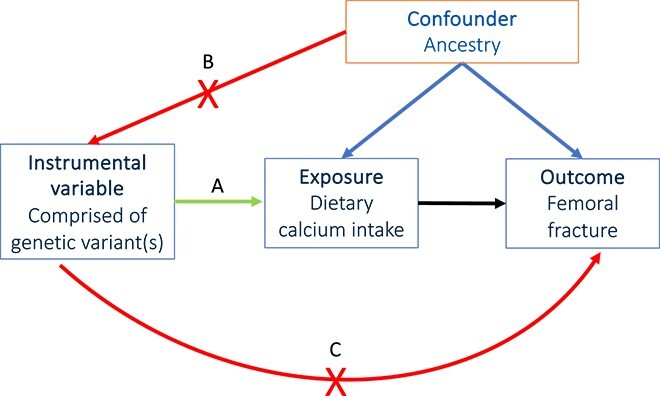
**Conditional assumptions in** MR**. (**A) Relevance: The genetic variant(s) acting as a proxy for the exposure variable are strongly associated with the exposure. (B) Independence: The IV is independent of confounding factors, thus genetic association with confounding variables is random, akin to randomization in an RCT. (C) Exclusion restriction: The IV affects the outcome solely through the exposure variable and not by external, horizontally-pleiotropic, pathways.

Mendel’s laws of genetic inheritance underpin the principles of MR.^8^ The law of “independent segregation” states that at every site on an autosome, 1 allele is inherited from the mother and 1 allele from the father, and which allele inherited from each parent is random.^9^^,^^10^ The law of “independent assortment” states that inheritance of alleles is independent of other inherited alleles or traits, except for genomic regions that are inherited together, known as linkage disequilibrium (LD) (Table 1).^9^^,^^10^^,^^17^ Based on these principles, MR studies that use genetic variants as an IV for an exposure can avoid bias from confounding variables (Table 1). To reliably draw causal inference from MR, 3 conditions must be met, and authors of MR studies should, as far as possible, demonstrate that these assumptions have not been violated (Figure 3):

Condition 1- Relevance: The IV must be reliably associated with the exposure.

Condition 2- Exchangeability or independence: The exposure IV is not caused by a factor that also causes the outcome, that is, no unmeasured confounding variables between the exposure IV and the outcome.

Condition 3- Exclusion restriction: The exposure IV only affects the outcome via the exposure trait and not by any other trait with a downstream effect on the outcome of interest.

Furthermore, robust MR findings rely on an assumption of gene–environment equivalence, that is, a change in an exposure due to genetic variation has the same downstream effects on a phenotype as an environmental or pharmaceutical influence.^18^ Not all genetic variation exactly mimics environmental alterations thus gene–environment equivalence must be considered when interpreting the reliability of results from MR.^16^ For example, genotypic influences on serum phosphate concentration or a similar increase in serum phosphate concentration influenced by dietary intake or supplementation would be expected to result in the same effects on reducing risk of kidney stone disease.^19^ Although there are not yet observational data that corroborate this finding, a meta-analysis showed that increasing phosphate intake lowers urine calcium, which provides preliminary triangulating evidence that higher phosphate might lead to a lower risk of kidney stones.^20^ While gene–environment equivalence cannot be “proven,” it is essential that researchers appraise whether it is a reasonable assumption considering available evidence regarding the biological mechanisms underlying the exposure-outcome relationship. For example, MR of lipid-lowering therapies such as statins (instrumented by genetic variants associated with the gene encoding HMG-CoA reductase) or PCSK9 inhibitors provides empirical evidence of gene–environment equivalence: MR yields genetically predicted causal effects that triangulate with findings from large phase III trials.^21^ A nuance is that the long, subclinical phase of atherosclerosis preceding symptomatic cardiovascular disease means that MR estimates of effect are much larger than those from shorter clinical trials. Understanding the scaling factors for each exposure-disease pair can help scientists to consider what results could be expected from a clinical trial.

Another important consideration in MR studies is the temporality of exposure. Using genetic variants inherited at conception as IVs means that MR study results reflect a lifelong exposure. For example, MR analysis of blood pressure on coronary artery disease represents years of exposure to hypertension rather than the follow-up period of an RCT designed to evaluate the effects of anti-hypertensive medication on coronary artery disease. Moreover, such studies assume that associations of genetic variants with an exposure are constant,^22^ however effects of an exposure on an outcome may relate to the level of the exposure at a certain stage in life. For example, evidence suggests that early life adiposity influences risk of breast cancer independent of later life adiposity.^23^

When using MR, it is important that the IV is derived from the same underlying population in which the outcome is assessed.^16^^,^^24^ For example, causal inference would be invalid if a study investigated the effect of alcohol intake using an exposure IV derived from a population of individuals who consume alcohol with an outcome IV derived from a population of individuals with little or no alcohol intake, for example women in some east Asian cultures.^25^ Considering genetic ancestry is crucial as patterns of LD vary across ancestral groups, hence if different ancestral groups are employed in MR analyses, a genetic variant may not have the same association with an exposure trait in exposure and outcome datasets.^26^ Reliance on ancestry-specific GWASs, combined with the current Eurocentric focus and lack of well-powered studies in non-European populations, exacerbates historic health research disparities and limits generalizability across populations.^27^

Further assumptions made in MR studies are that genetic instruments demonstrate linear associations with an outcome and are homogeneous, that is, the relationship between an IV and exposure is the same throughout the studied population.^28^

## MR study design

MR can be undertaken using either individual-level genetic and phenotype data or from GWAS summary statistics. The latter allows researchers to consider larger sample sizes and more easily share data across institutions. As sample size, case–control ratio, and variance explained by IVs increase in a GWAS with a binary endpoint, so does the statistical power and precision of the study.^16^ Individual genetic variants may be used as discrete instruments or combined into an allele score generated from the sum of risk alleles.^29^ Allele scores should be weighted to reflect the estimated effect of each variant on an exposure to account for different variant-effect associations. Combining appropriately-selected variants into an allele score can increase the power of analyses which is particularly useful when individual variants have a small effect on the exposure, represented by the beta-coefficient or odds-ratio, and using individual variants could hypothetically violate the relevance assumption of MR (weak instrument bias, Table 1).^29^ Using multiple variants in MR is akin to combining data from multiple trials; where multiple variants are used it is important that these are independent (ie, not in LD) to prevent the same association signal being considered twice. Therefore, prior to performing MR, variants in an exposure IV that are in LD should be “clumped” to retain only 1 variant per locus, using a suitable population linkage pattern. Alternatively, an LD matrix can be included in analyses to account for correlation between variants.^30^

Because common genetic variants typically account for a small proportion in the variation of a trait, large GWAS datasets are frequently used to derive well-powered MR studies. Multiple consortia and GWAS catalogs provide publicly-available summary statistics from GWAS and meta-analyses that facilitate these analyses. Supplementary Table S1 details some large GWAS consortia and catalogs used in MR studies.^6^^,^^31–42^ For example, the GEnetic Factors for OSteoporosis Consortium (GEFOS) is an international collaboration that used data from 20 439 fracture cases and 78 843 controls to identify risk factors for osteoporotic fractures using summary MR.^43^

### One-sample MR

One-sample MR refers to analyses performed using data from a single population. Estimates derived from 1-sample analysis may be subject to overfitting, that is, bias away from the null hypothesis, leading to an incorrect assumption of causality.^11^^,^^29^^,^^44^ The risk of overfitting arises because the exposure IV is used to predict the outcome in the same population it was derived from, risking chance correlations with potential confounding variables.^45^ This can be particularly problematic in the presence of weak instrument bias, an issue that can limit both 1- and 2-sample MR studies. However, as MR has advanced and researchers now use stronger instruments from large consortia and databases, the risk of overfitting has decreased.

### Two-sample MR

Two-sample MR analyses assess the effect of an exposure on an outcome in a dataset that is different from that in which the exposure IV was derived. This has particular utility when a dataset is limited by small sample size or a lack of appropriate biospecimens; researchers can leverage larger datasets for genetic associations with the exposure and outcome separately, which can improve the statistical power of analysis.^11^ Two-sample MR assumes that the populations from which the IV associations are derived share equivalent genetic ancestry, age, and sex distributions.^26^^,^^46^ It is important that there is adequate case ascertainment in the population from which the outcome IV is derived to ensure that MR estimates are well-powered. Increasing use of GWAS from biobanks and consortia can result in sample overlap in 2-sample MR.^46^ The effect of overlap on a continuous outcome is proportional to the degree of overlap between samples; as overlap increases the study design more closely resembles that of 1-sample MR. For binary outcomes the effects of overlap depend on whether case participants’ data have been used to derive exposure IV^45^; if only control participants overlap between exposure and outcome datasets then unbiased estimates are achieved, however if both case and control participants overlap then the effect of bias is comparable to that on a continuous outcome.^45^ Recent analyses propose that sample overlap may not be as problematic as previously suggested. Power, bias, and type 1 error rate in the context of sample overlap can be estimated using tools such as the web application https://sb452.shinyapps.io/overlap.^45^

## MR estimates

### Wald ratio

The simplest method for deriving an estimate for the causal effect of a single IV on an outcome is the Wald ratio (Table 1). This is the ratio of the regression of the outcome on the IV and the regression of the exposure on the IV.^47^ For continuous outcomes, the resulting estimate is typically expressed as the change in outcome per unit change in exposure due to the variant of interest. For binary outcomes, the Wald ratio is interpreted as the log odds ratio for disease risk per unit increase in the exposure due to the variant of interest. A Wald ratio intimates that a variant may exert causal effects on an outcome; however, one must be mindful that the variant may be in LD with an outcome-associated variant, an issue that can also affect an IV comprised of multiple genetic variants, and may lead to erroneous conclusions.

### Inverse variance weighted estimates

To obtain an overall MR estimate for an IV comprising multiple variants, researchers often conduct an inverse variance weighted (IVW) meta-analysis of each genetic variant’s Wald ratio (Table 1). Conceptually, the IVW model can be considered as a regression of variant-outcome effects against variant-exposure effects weighted according to the inverse of the variance of the variant-outcome effects. The intercept of this regression is fixed at zero (ie, a variant with no effect on the exposure has no effect on the outcome).

A fixed effect IVW is calculated assuming that all variants within the IV uphold the assumptions of MR. When all MR assumptions are met, the IVW estimate has the highest statistical power and is often used alongside other MR methods as sensitivity analyses.^48^ However, should any variants in the IV exhibit horizontal pleiotropy (Table 1), that is, they influence the outcome via pathways other than the exposure of interest, then the IVW estimate may be biased.^48^ Balanced horizontal pleiotropy (the deviation of a variant’s estimate from the mean estimate is independent of its effect on the exposure) may be accounted for if the random effects do not systematically affect the outcome. Here, the IVW method can provide an unbiased estimate of causal effect.^49^

### MR-Egger estimates

If there is directional horizontal pleiotropy and variants in an IV affect the outcome through pathways not mediated by the exposure (Table 1, arrow C Figure 3), the IVW estimate is biased and the assumption of exchangeability is violated. This can be overcome by enabling a “non-zero” intercept when regressing variant-outcome effects against variant-exposure effects, allowing for directional (ie, “unbalanced”) horizontal pleiotropy (Table 1).^50^^,^^51^ In MR-Egger, the intercept estimate represents the average pleiotropic effect across all variants and the slope is an estimate of the causal effect after accounting for pleiotropy.

The MR-Egger estimate can be unbiased even if all variants in an IV exhibit horizontal pleiotropy provided that the effects of horizontal pleiotropy are not related to variant-exposure effects (the InSIDE assumption (Instrument Strength Independent of Direct Effect)). For example, if a study investigates the relationship between stress levels and the risk of coronary heart disease and variants in the exposure IV are associated with smoking, it is plausible that these variants affect risk of heart disease by influencing both smoking and stress levels. If the genetic effects on smoking behaviors are correlated with the effects of genetic variants on stress levels then the InSIDE assumption is violated and the MR-Egger estimate will be biased.^52^ Moreover, MR-Egger estimates are unreliable when there are few genetic variants in an IV because the precision of the estimate decreases as the variability of variant-exposure associations increases.^50^

### Weighted median

An alternative method of addressing unbalanced pleiotropy is to use the median effect of all variants within an IV (Table 1).^53^ Whereas the simple median estimate provides a consistent causal estimate if half of variants in an IV are valid, the weighted median provides a consistent estimate when at least half of the weight comes from valid variants that do not violate the assumptions of MR. Variants are weighted by the inverse variance of their association with the outcome; thus, variants with stronger associations contribute more to the weighted median estimate.

### Simple mode and weighted mode

Mode-based estimates generate clusters of variants, using a kernel density function, with each group having similar causal effects (Table 1). The resulting estimate is derived from the cluster comprising the most variants.^54^ If the largest cluster comprises valid variants, the mode-based estimate will be unbiased. A weighted estimate involves weighting each variant’s contribution to its cluster according to the inverse variance of its association with the outcome.

### MR pleiotropy residual sum and outlier

The MR-pleiotropy residual sum and outlier (MR-PRESSO) method aims to identify horizontally pleiotropic outliers to overcome bias that arising from MR estimates influenced by horizontal pleiotropy (Table 1).^55^ This method applies 3 sequential tests to summary-level MR: identifying horizontal pleiotropy (global test), correcting pleiotropy by removing outlying variants (outlier test), and ascertaining whether there are significant differences in causal estimates after outlying variants have been removed (distortion test).^55^

### Multivariable MR

It may be relevant to undertake multivariable analysis to ascertain the effect of an exposure on an outcome after controlling for relevant covariates (Table 1). Multivariable MR is of particular utility when the “*exclusion restriction*” assumption has been violated, as it allows genetic variants to have pleiotropic effects on the outcome via other risk factors.^56^ Furthermore, multivariable MR allows multiple, correlated, exposures to be included in analyses, and enables researchers to ascertain if all included exposure variables exert direct effects on an outcome and to identify mediating relationships providing that the F-statistic (see “Assessing the Core Assumptions of MR” section) is sufficiently high and thus there are sufficient genetic differences between included traits. Multivariable MR has been used to allude to the role of adiposity and risk of kidney stone disease as risk of kidney stone disease increases with higher BMI, independent of waist-to-hip-ratio (WHR), and with higher WHR independent of BMI.^19^

Multivariable MR relies on the same assumptions as univariable MR.^57^ Furthermore, multivariable MR assumes that the included genetic variants affect the outcome only via the risk factors included in analysis.^56^

### Mediation MR

**Figure 4:**
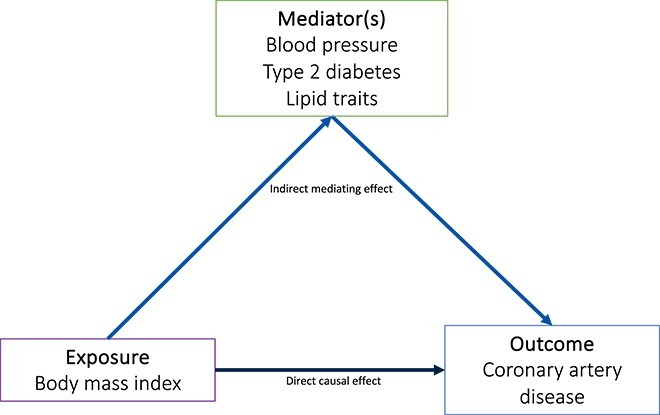
**Mediation** MR**.** Mediation analysis estimates the effect of an exposure on an outcome via mediating factors (indirect mediating effect) and via mechanisms independent of the mediating factor (direct casual effect). The proportion mediated effect can subsequently be derived.

Mediation analyses enable exploration of the pathways by which an exposure affects an outcome, and estimation of the proportion of the effect of the exposure on the outcome mediated by an additional trait (Figure 4, Table 1).^58^

Mediation MR analyses can provide an estimate of the effect of an exposure on an outcome via all possible pathways (total effect), an estimate of the effect of an exposure on the outcome via mechanisms independent of the mediating factor(s) (direct effect), and an estimate of the effect of the exposure on the outcome via the specified mediator(s) (indirect effect).^58^ These analyses have been used to demonstrate that higher WHR mediates 12%-15% of its effect on kidney stone risk through increasing serum calcium concentrations,^19^ and that higher WHR and BMI increase the risk of coronary artery disease via effects mediated by systolic blood pressure (~27%), type 2 diabetes (~41%), and lipid traits (~3%).^59^

Mediation analysis can be undertaken either using 2-step or multivariable MR methods. Using 2-step MR, the effect of the exposure on the mediator and the effect of the mediator on the outcome can be multiplied to ascertain the indirect effect (product of coefficients method).^60^ In multivariable MR methods, the direct effect of the exposure on the outcome, controlling for the mediator, is estimated. The indirect effect is calculated by subtracting the“controlled” direct effect from the total effect (difference method).^60^ The “proportion mediated effect” can be derived to estimate the proportion of the total effect of an exposure on an outcome explained by a mediating pathway (Figure 4).

### Non-linear MR

If associations between an exposure and outcome are more complex than a linear function, MR results from parametric methods, including IVW, MR-Egger, mode-, and median-based estimates, will be unreliable; non-linear MR methods may confirm or refute a causal effect (Table 1),^61–63^ where there is a relationship between an exposure and outcome. Moreover, non-linear MR analyses can identify population subsets that are more vulnerable to disease.

For example, a (now withdrawn) study of serum 25-hydroxyvitamin-D concentrations and cardiovascular outcomes and mortality suggested that increasing vitamin-D concentrations in populations with low vitamin-D concentrations may decrease mortality.^64^ However, subsequent analyses in the same population revealed that the constant genetic effect assumption had been violated.^65^ When the authors reanalyzed data using the non-parametric stratification “doubly ranked” method, which does not rely on the constant genetic effect assumption, no evidence of a link between vitamin-D and mortality at any vitamin-D concentration was identified.^61^

Conversely, observational analyses have described a J-shaped relationship between BMI and all-cause mortality.^66^ A causal effect of BMI on all-cause mortality was shown in linear and non-linear MR estimates although the J-shaped curve was flatter in non-linear MR estimates than in conventional, observational, epidemiological analyses. Following meta-regression analyses, authors demonstrated that the non-linear relationship was driven by extreme quantiles of BMI where datapoints were scarce. After removing the first and 99th percentiles of BMI data MR estimates indicated a linear effect of BMI on mortality.^66^

Recent analyses have discussed problems related to non-linear MR methods.^67^ Wade et al. adopted a negative control methodology to evaluate these methods. Their results that higher BMI increases the odds of being of male sex highlight the biases of residual and doubly-ranked based approaches. In the same candid article the authors express concern over the reliability of results in publications drawing conclusions from non-linear MR analyses including their own “>70 other papers”. Overall, while MR is a decades-old field, there remain complexities in reliably instrumenting polygenic traits.

## Drug-target MR

MR has been applied to predict drug effects and toxicities in humans^68^ and provide evidence for the likely utility of a drug prior to embarking on costly clinical trials. Assuming that a drug-target is a protein, MR can be employed to proxy effects of targeting this protein by using genetic variants that are a valid IV of the target protein and estimating the effect of modulating this protein on an outcome. Restricting the IV to variants within a defined distance of transcription start and stop sites (cis protein quantitative loci) increases confidence that identified effects are exerted via the gene encoding the protein.^69^ Many drug-target MR analyses triangulate evidence, including genetic variants associated with a biomarker affected by a drug’s target. Drug-target MR estimates the effect of changing this biomarker, by altering the drug target’s activity, on an outcome. For example, statins and proprotein convertase subtilisin/kexin type 9 (PCSK9) inhibitors are used to reduce low-density lipoprotein cholesterol (LDL-c) to prevent cardiovascular disease.^70^^,^^71^ However, lower LDL-c is associated with a higher incidence of type 2 diabetes.^72–74^ Schmidt et al. identified variants at the *PCSK9* locus associated with reductions in LDL-c and demonstrated that lower LDL-c results in higher body weight, WHR, fasting plasma glucose, HbA1c, and liability to type 2 diabetes.^75^ As previously mentioned, the magnitude of effect from a MR study may not mirror that of a clinical intervention as MR estimates represent life-long exposure. Furthermore, causal estimates derived from MR do not necessarily mirror the effects seen in clinical practice, as demonstrated by the hitherto lack of effect of PCSK9 inhibition on increasing the risk of type 2 diabetes in RCTs.^76–79^

A further use of MR in drug-development is through phenome-wide association studies (PheWAS) to explore a wide range of effects of modulating drug targets.^80–83^ Wang *et al.* used a genetic proxy of angiopoietin-like proteins 3 and 4 (*ANGPTL3* and *ANGPTL4*) and lipoprotein lipase (*LPL*) to demonstrate an association with lipoprotein lipids and fatty acids, and employed drug-target MR to reveal that modulating coronary heart disease and type 2 diabetes risk at the *ANGPTL4* and *LPL* loci was predicted to be clinically beneficial with concomitant predicted effects on WHR, whereas an *ANGPTL3* variant showed a distinct effect on renal function.^84^ These findings replicated those reported in clinical studies.^85–88^

## Visual representation of MR analyses

**Figure 5:**
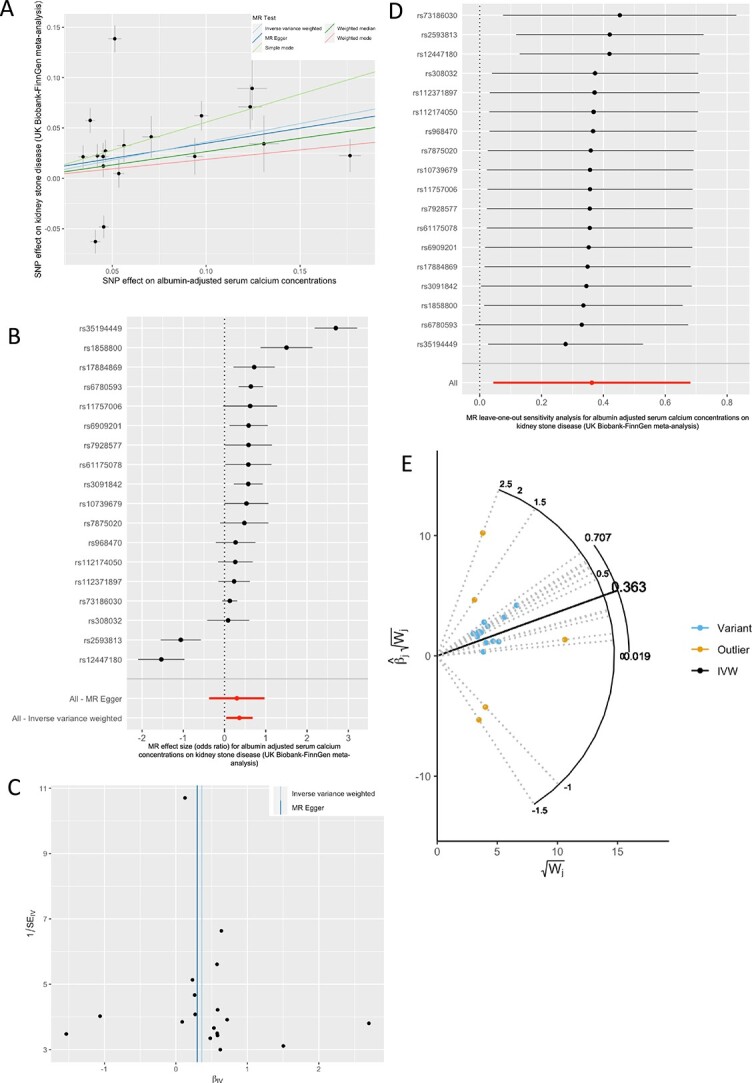
**Visual representations of** MR **analyses of the effect of albumin-adjusted serum calcium concentrations on risk of kidney stone disease- reanalysis of data from Lovegrove et al.**^19^ MR, Mendelian randomization; SNP, single nucleotide polymorphism. (A) Scatter plot of genetic associations and causal estimates. (B) Forest plot of variant-specific causal estimates, IVW, and MR-Egger estimates. (C) Funnel plot of variant-specific causal estimates. The estimate effect is plotted against the precision (reciprocal of standard error) of the estimate for each genetic variant. The vertical lines correspond to the estimates for IVW and MR-Egger estimates. (D) Forest plot of leave one out analyses. For each variant, the effect of removing it from the IV is plotted. (E) Radial plot. The x-axis represents the weigh attributed to each genetic variant and the y-axis represents its Z-statistic (point estimate divided by standard error). The greater the vertical distance between a genetic variant and the overall causal estimate, the greater its contribution to Cochran’s Q statistic of heterogeneity.

There are multiple methods to visualize MR findings, each providing specific information to facilitate assessment of the reliability of a study and its underlying assumptions.^19^ A common representation of summary-data MR is a scatter plot of associations of individual variants with the exposure and outcome (Figure 5a). Error bars, representing the standard error of the estimate in the exposure and outcome, surround each point and regression lines for each sensitivity analysis may be overlayed showing the overall estimate of the analysis. Scatter plots reveal whether variants have similar estimates of effect or if there is heterogeneity and the overall estimate is driven by a single genetic variant (Figure 5a). Individual variant-specific causal effects can be plotted as a forest plot and compared with overall IVW and MR-Egger estimates (Figure 5b). Both scatter and forest plots can help to identify heterogeneity in the causal estimates from different variants.^26^ Funnel plots enable assessment of asymmetry in causal estimates and directional pleiotropy if estimates from weaker genetic variants are biased in 1 direction (Figure 5c).

Leave-one-out analysis plots represent the effect of removing 1 genetic variant at a time on the overall IVW MR estimate, enabling one to identify a genetic variant with an important influence on the overall result (Figure 5d). Using this information, researchers can assess how robust their findings are to removing these variants.

Radial MR plots enable one to visualize the weight attributed to each variant included in the MR estimate and identify outliers (Figure 5e).^89^ The distance between each point and the IVW slope represents the contribution of the genetic variant to Cochran’s Q-statistic of heterogeneity.^89^

## Assessing the core assumptions of MR

**Figure 6:**
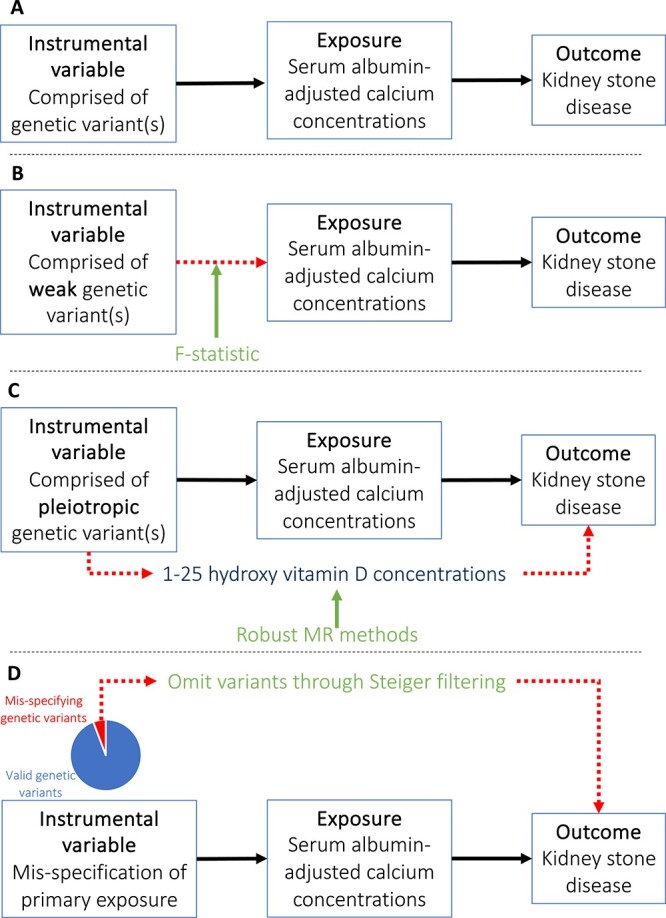
**Assessing assumptions in** MR. (A) Assumption in MR: MR assumes that there is an IV comprised of genetic variant(s) which are a proxy of the effect of an exposure variable in an outcome measure. (B) The F-statistic can evaluate the strength of association between the IV and exposure variable. (C) Robust MR methods, for example MR-Egger regression, can identify when genetic variants have pleiotropic effects and produce a reliable effect estimate so long as the magnitude of pleiotropic effects is not correlated with the variant-exposure effects. (D) Steiger filtering selects genetic variants that explain more variance in the exposure variable than the outcome and excludes those that fail to meet this condition to mitigate against mis-specification of the primary exposure.

Whereas combining genetic variants into an IV increases power, there is a risk of “weak instrument bias” because each variant has a small effect and may violate the “relevance” assumption. Weak instruments reduce the power to detect a causal effect and may result in a biased MR estimate.^45^ In 1-sample MR this estimate is biased toward identifying a relationship between the risk factor and outcome, whereas in 2-sample MR it is biased toward the null.^46^ Careful selection of genetic variants for IVs, for example ensuring that variants reach GWAS-significance (*p*<5 × 10^-8^), can reduce bias from a weak instrument. The F-statistic quantifies the strength of an IV by regressing an exposure on a set of variants (Figure 6b).^47^ The higher the F-statistic, the more robust the instrument; a score greater than 10 indicates that the IVs are sufficiently strong for univariable and multivariable MR analyses.^57^ An F-statistic less than 10 does not confirm that an instrument is insufficiently strong but indicates that researchers should consider the possibility of weak instrument bias within their analyses.

If all genetic variants are valid instruments, one would expect the relationship between variants, the exposure, and outcome to be linear and homogenous for all individuals within a population, and for there to be no more heterogeneity in variant-exposure and variant-outcome estimates than explainable by chance alone.^26^ However, this theory is seldom, if ever, satisfied. Heterogeneity may arise from variants acting on a trait through a variety of physiological mechanisms or from non-linear^72^ relationships between variants, exposures, and outcomes within a population. Heterogeneity can be calculated for IVW and MR-Egger estimates using Cochrane’s Q or the I^2^ statistic which tests for horizontal pleiotropy (Figure 6c).^54^ Quantifying heterogeneity enables researchers to identify outliers and mitigate for horizontal pleiotropy, for example by employing MR-PRESSO.^26^ Heterogeneity can be visualized using scatter, forest, funnel, or radial plots reflecting the Wald ratio of each variant within an IV (Figure 5).^46^

Variants should explain more variance in the exposure trait than the outcome and the estimated effect of the IV on the outcome should be wholly mediated through the exposure.^26^ Measurement error, sample size discrepancies between phenotypes, or a combination of quantitative and binary phenotypes can cause a genetic variant to seemingly explain more variation in a more distal trait than a more proximal trait.^90^ For example, 1,25-hydroxyvitamin-D influences albumin-adjusted serum calcium concentrations; if a genetic variant primarily associated with 1,25-hydroxyvitamin-D is included within an IV for albumin-adjusted serum calcium concentrations then misleading estimates for the causal effect of albumin-adjusted serum calcium concentrations on a range of outcomes may arise. To mitigate against this problem and address possible misspecification of the primary exposure “Steiger filtering” or “MR Steiger” can be performed (Figure 6d).^91^ This entails removing genetic variants that explain more variation in the outcome than the exposure and assessing the impact of this process on the MR estimate.^90^ MR Steiger can provide evidence supporting the direction of a causal relationship and performing bidirectional MR (analyzing the effect of the exposure on the outcome, and the effect of the outcome on the exposure) using separate sets of IVs can also assess the direction of causal effects.

Using positive and negative control outcomes can be valuable when assessing how robust an MR analysis is.^26^ A positive control outcome is one known to be caused by the exposure variable; for example, systolic blood pressure is known to increase risk of stroke.^92^^,^^93^ It is logical that genetic variants associated with systolic blood pressure will reveal that higher systolic blood pressure increases risk of stroke on MR analyses; if the analyses failed to identify this association, this may indicate that the genetic variants are not valid instruments to proxy systolic blood pressure. Conversely, a negative control outcome is one not understood to be caused by the exposure variable.^26^^,^^94^ Thus, genetic variants associated with coffee consumption may have causal effects on risk of heart disease but would not be expected to affect whether people have blue eyes. In this example, eye color acts as a negative control; if the MR estimate shows a significant relationship between the IV proxying coffee consumption and risk of heart disease but not on eye color it would support the validity of the IV and the integrity of the resulting estimate. Conversely, were the MR estimate to show a relationship between the IV for coffee consumption and blue eye color, one should be suspicious of bias, population stratification, or pleiotropy within the IV.

## MR in bone and mineral research

To contextualize the possible application of MR to bone and mineral research for readers, we have identified a small selection of studies in the field:

### BMD and osteoarthritis

Observational analyses indicate that high BMD is a risk factor for osteoarthritis.^95^ Hartley et al. used MR to ascertain whether this was a causal relationship, if the effect was unidirectional, and whether the relationship was independent of BMI.^96^ Findings suggested that effects were bidirectional, and that increasing heel BMD increased liability to knee and hip osteoarthritis. Multivariable MR indicated that there is a BMI-independent pathway by which higher BMD causally increases liability to osteoarthritis.^96^

### Serum calcium and BMD

Cerani *et al.* aimed to determine whether higher serum calcium concentrations are linked to higher BMD and protection against osteoporotic fractures.^97^ Using summary statistics from a serum calcium concentrations GWAS and a GWAS meta-analysis of fractures including data from GEFOS they found no evidence for an effect of serum calcium on BMD or fracture risk.^43^^,^^98^ The authors acknowledge that the study may not reflect the effect of calcium supplementation on these outcomes. These results align with those of Trajanoska *et al.* who evaluated the role of 15 risk factors, including estimated calcium intake, on liability to osteoporotic fracture using MR and concluded that BMD was the only one of the evaluated risk factors to show a causal effect.^43^ Moreover, findings from MR studies corroborate with those from the RECORD (Randomized Evaluation of Calcium Or vitamin D) RCT which did not show that routine oral supplementation with calcium reduces fracture risk.^99^

### SGLT2-inhibition and BMD

Finally, Dai *et al.* used drug-target MR to ascertain whether there may be a beneficial effect from sodium-glucose cotransporter 2 (SGLT2) inhibition on BMD, risk of osteoporosis, or fracture.^100^ SGLT2 inhibitors have been employed as anti-diabetic agents and have proven to be beneficial in patients with cardiac or renal failure regardless of diabetic status; however, concern exists regarding increased fracture risk.^101–105^ The authors constructed an IV for SGLT2 inhibition based on genetic variants that affect *SLC5A2* expression, have regional-wide association with HbA1c (*p*<1x10^−4^), and explain genetic colocalization between SLC5A2 expression and HbA1c concentrations.^100^ Using this IV, Dai et al. found no evidence for a causal effect of SGLT2 inhibition on fracture risk and recommend that these results can be used to inform guidelines for patients prescribed this class of medications. Their results support those of RCTs reporting no effect of SGLT2 inhibitors on markers of bone formation, resorption, BMD, or fracture risk.^106–110^

## Software in MR

Multiple MR software packages are at researchers’ disposal and can be implemented in standard statistics packages, for example R and Stata. Software packages typically include methods to derive several MR estimates, perform sensitivity analyses, and assumption testing. Software packages for MR also enable researchers to derive graphs as a visual representation of MR results. The MendelianRandomization package, implemented in R, can extract summarized data related to associations with named exposure and outcome variables from PhenoScanner (http://www.phenoscanner.medschl.cam.ac.uk/), a curated database of publicly-available results from large-scale GWAS, hosted by the University of Cambridge.^111^^,^^113^ Similarly, the TwoSampleMR package, also implemented in R, enables researchers to derive exposure or outcome IVs from the University of Bristol’s Integrative Epidemiology Unit Open GWAS Project (https://gwas.mrcieu.ac.uk/) comprising a manually-curated collection of complete GWAS summary datasets, available as open source files, that can be queried or downloaded.^31^^,^^49^^,^^90^ The same group have also published MR-Base (https://app.mrbase.org/), a web app aiming to simplify MR implementation.^29^^,^^45^ As well as searching for genetic variants across multiple GWAS, it can perform 2-sample MR, sensitivity analyses, and produce MR visualization plots.

## Reporting guidelines in MR studies

In 2021, the “STrengthening the Reporting of OBservational studies in Epidemiology- Mendelian randomization” (STROBE-MR) guidelines were published, providing MR studies with reporting guidelines similar to those already in existence for cohort, case–control, and cross-sectional studies.^1^^,^^15^^,^^114^ Akin to the original STROBE checklist, STROBE-MR recommends using a 20-point checklist (available at https://www.strobe-mr.org/) to enable readers, journal reviewers, and editors to evaluate the quality of an MR study. The checklist is divided into sections of a paper (Title and abstract; Introduction; Methods; Results; Discussion; Other information) with opportunity for authors to detail the page location in their manuscript and the specific text that addresses the relevant checklist item.

## Future directions: pragmatic use of MR studies

MR offers many practical advantages over traditional observational and interventional studies, for example deriving results rapidly rather than waiting for months or years for outcomes to arise in conventional epidemiological research, and the facility to draw on large, publicly-available GWAS datasets to inform clinical trials, thus improving the cost-efficiency of primary research in healthcare. However, caution is needed to ensure that the findings from MR studies are interpreted appropriately (Box 1).

Box 1. Considerations when appraising MR literature.

Study design:

- Is there a logical rationale for the study?
- Might the study contribute to the scientific literature?

Instrumental variables:

- Are the data from which IVs have been derived relevant, well-powered, and of a suitable genetic ancestry?
- What *p*-value threshold has been used for inclusion of genetic variants in IVs?
- Has linkage-disequilibrium (LD) been accounted for (eg, by clumping variants or incorporating an LD matrix)?
- How strong is the IV (F-statistic, R^2^)?

Analyses:

- Have a range of sensitivity analyses been considered including those that address pleiotropy?
- Is there consistency in results across sensitivity analyses?
- Have authors corrected for multiple testing?
- Have reasonable efforts been made to demonstrate that the 3 core assumptions (*relevance, independence, exclusion restriction*) of MR have not been violated?

Interpretation:

- Have results been triangulated with other data (eg, eQTL or pQTL data) and other study designs (conventional observational, interventional, in vitro*,* in vivo*, in silico*)?
- Do the conclusions align with the data that have been presented?

Beyond appraising the methods, discussed in this review, one should triangulate MR analyses results, evaluating and contextualizing the place that they have amidst evidence from other sources. Care should be taken to ensure that the MR study is testing a biologically-plausible hypothesis; IVs are valid genetic proxies; included data are derived from phenotypes, interventions, and study populations reflecting the underlying research question (Box 1)^6^; MR analyses are reported in a reproducible manner; and that findings from other study designs support the arising MR results, or vice versa.^115^ Just as researchers critically appraise observational and interventional studies, the same rigor should be applied to the MR literature; STROBE-MR guidelines can be beneficial in this regard.^14^^,^^15^

## Conclusions

MR studies can complement conventional epidemiological methods to enable researchers to infer causal effects in health and disease. Novel approaches to MR analyses are actively evolving and can provide insights into disease pathophysiology by overcoming many of the limitations that surround observational and interventional methods. However, despite the benefits that MR can confer, MR is subject to limitations and evaluating the assumptions made is crucial when performing and interpreting MR studies.

## Supplementary Material

Supplementary_table_1_zjae136

## Data Availability

Data sharing not applicable to this article as no datasets were generated or analyzed during the current study.

## Rights retention statement

For the purpose of Open Access, the author has applied a CC BY public copyright license to any Author Accepted Manuscript (AAM) version arising from this submission.
