# Supplementary material for: Causal inference in health and disease: a review of the principles and applications of Mendelian randomization
Source: J Bone Miner Res. 2024 Aug 21;39(11):1539–52. doi: 10.1093/jbmr/zjae136 (PMC11523132; doi:10.1093/jbmr/zjae136)
Supplement: Supplementary_table_1_zjae136 [file supplementary_table_1_zjae136.docx]

Supplementary Table 1. Examples of some large genome-wide association study consortia

| **Catalogue** | **Description** | **Access** |
| --- | --- | --- |
| GWAS Catalog^1^ | National Human Genome Research Institute GWAS catalog | https://www.ebi.ac.uk/gwas/ |
| MRC IEU OpenGWAS^2^ | A manually curated collection of complete GWAS summary datasets made available as open-source files for download | https://gwas.mrcieu.ac.uk/ |
|  | | |
| **Consortium** | **Description** | **Access** |
| CARDIoGRAMplusC4D^3^ | (Coronary ARtery DIsease Genome wide Replication and Meta-analysis (CARDIoGRAM) plus The Coronary Artery Disease (C4D) Genetics) consortium | http://www.cardiogramplusc4d.org/ |
| DIAGRAM consortium^4^ | DIAbetes Genetics Replication And Meta-analysis | https://diagram-consortium.org/ |
| EGG^5^ | Early Growth Genetics Consortium. Data from multiple studies on human traits related to early growth | http://egg-consortium.org/ |
| GEFOS^6^ | GEnetic Factors for OSteoporosis Consortium | http://www.gefos.org/ |
| GIANT^7^ | Genetic Investigation of ANthropometric Traits | https://portals.broadinstitute.org/collaboration/giant/index.php/GIANT_consortium |
| GLGC^8^ | Global Lipids Genetics Consortium. World-wide collaboration of investigators dedicated to understanding the genetic aetiology of quantitative lipid traits. | http://www.lipidgenetics.org/ |
| ICBP^9^ | International Consortium for Blood Pressure Genome-Wide Association Studies | https://www.ncbi.nlm.nih.gov/projects/gap/cgi-bin/study.cgi?study_id=phs000585.v1.p1 |
| IIBDGC^10^ | International Inflammatory Bowel Disease Genetics Consortium | https://www.ibdgenetics.org/ |
| MAGIC^11^ | Meta-Analyses of Glucose and Insulin-related traits Consortium | http://magicinvestigators.org/ |
| Psychiatric Genomics Consortium^12^ | Uncovering the role of genetics in psychiatric disorders | https://pgc.unc.edu/ |
| SSGAC^13^ | Social Science Genetics Association Consortium | https://www.thessgac.org/ |

1. Sollis E, Mosaku A, Abid A, et al. The NHGRI-EBI GWAS Catalog: knowledgebase and deposition resource. *Nucleic Acids Res*. 2023;51(D1):D977-D985. doi:10.1093/nar/gkac1010

2. Elsworth B, Lyon M, Alexander T, et al. *The MRC IEU OpenGWAS Data Infrastructure*. Genetics; 2020. doi:10.1101/2020.08.10.244293

3. CARDIoGRAMplusC4D - CARDIoGRAMplusC4D Consortium. Accessed November 28, 2023. http://www.cardiogramplusc4d.org/

4. DIAGRAM Consortium. Accessed November 28, 2023. https://diagram-consortium.org/index.html

5. EGG (Early Growth Genetics) Consortium. Accessed November 28, 2023. http://egg-consortium.org/

6. Home. Accessed November 28, 2023. http://www.gefos.org/?q=home

7. GIANT consortium - Giant Consortium. Accessed November 28, 2023. https://portals.broadinstitute.org/collaboration/giant/index.php/GIANT_consortium

8. GLGC. Accessed November 28, 2023. http://www.lipidgenetics.org/

9. dbGaP Study. Accessed November 28, 2023. https://www.ncbi.nlm.nih.gov/projects/gap/cgi-bin/study.cgi?study_id=phs000585.v2.p1

10. Tastad C. IIBDGC. IIBDGC. Accessed November 28, 2023. https://www.ibdgenetics.org/

11. Magic Investigators - Home Page. Accessed November 28, 2023. https://magicinvestigators.org/

12. PGC – Psychiatric Genomics Consortium. Accessed November 28, 2023. https://pgc.unc.edu/

13. Home. ssgac. Accessed November 28, 2023. https://www.thessgac.org
